# Morphological and Chemical Traits of *Cladonia* Respond to Multiple Environmental Factors in Acidic Dry Grasslands

**DOI:** 10.3390/microorganisms9020453

**Published:** 2021-02-22

**Authors:** Gabriele Gheza, Luca Di Nuzzo, Chiara Vallese, Matteo Barcella, Renato Benesperi, Paolo Giordani, Juri Nascimbene, Silvia Assini

**Affiliations:** 1 BIOME Lab, Department of Biological, Geological and Environmental Sciences, Alma Mater Studiorum–University of Bologna, Via Irnerio 42, 40126 Bologna, Italy; gheza.gabriele@gmail.com (G.G.); chiara.vallese2@unibo.it (C.V.); 2 Department of Biology, University of Florence, Via La Pira 4, 50121 Florence, Italy; luca.dinuzzo@unifi.it (L.D.N.); renato.benesperi@unifi.it (R.B.); 3 Department of Earth and Environmental Sciences, University of Pavia, Via S. Epifanio 14, 27100 Pavia, Italy; matteo.barcella@unipv.it (M.B.); silviapaola.assini@unipv.it (S.A.); 4 Department of Pharmacy, University of Genova, Viale Cembrano 4, 16148 Genova, Italy; giordani@difar.unige.it

**Keywords:** lichens, open dry habitats, reproduction strategy, secondary metabolites, species traits, thallus growth forms, vegetation dynamics

## Abstract

Terricolous lichen communities in lowlands occur especially in open dry habitats. Such communities are often dominated by species of the genus *Cladonia*, which are very variable in morphology, reproduction strategies, and secondary metabolites. In this work, we investigated traits-environment relationships considering vegetation dynamics, substrate pH, disturbance, and climate. A total of 122 plots were surveyed in 41 acidic dry grasslands in the western Po Plain (Northern Italy). Relationships between *Cladonia* traits and environmental variables were investigated by means of a model-based Fourth Corner Analysis. Thallus morphology and metabolites responded to vegetation dynamics, substrate pH, disturbance, and climate, whereas reproduction strategies responded only to vegetation dynamics. Traits’ correlations with vegetation dynamics elucidate their colonization patterns in open dry habitats or suggest biotic interactions with bryophytes and vascular plants. In addition, correlations between metabolites and environmental factors support interpretations of their ecological roles. Our results also stress the importance of studying traits’ relationships with climatic factors as an alert towards lichen reactions to climate change.

## 1. Introduction

The analysis of functional traits to explore species’ responses to environmental factors is increasingly applied also to lichens [1,2]. However, most studies have addressed epiphytes (e.g., [3,4,5]), whereas terricolous lichens are relatively less investigated (e.g., [6,7,8]) despite their ecological importance (e.g., [9]) and conservation concern [6,10]. It is therefore of utmost importance to understand the relationships driving terricolous species’ responses to environmental stresses, particularly in the current context of global change [2,5,6]

Terricolous lichen communities are often dominated by species of the genus *Cladonia*. In this genus, the thallus is composed of two parts: a basal primary thallus and a secondary thallus with a very variable morphology across species [11,12,13]. This high variability leads to a range of possible morphological combinations that have been almost overlooked in previous literature (e.g., [6,7,14,15]). Additionally, reproduction, which is a relevant trait in species life history [5,6], is achieved in variable ways, not only sexually (by means of ascospores), but also with different types of vegetative propagules [11,12], as well as by dispersion of thallus fragments [16]. The position of pycnidia may also vary, developing either on the primary or secondary thallus [12,13]. *Cladonia* lichens produce several secondary metabolites, some of them being widely studied due to their potential cytotoxic activity [17,18,19]. However, their relationships with environmental factors have been little explored [20,21,22].

In lowland landscapes of central-southern Europe, *Cladonia*-dominated communities occur especially in open dry habitats on acidic substrates [10,14,23,24,25,26]. Open dry habitats are among the most threatened by human activities—land-use change, land consumption, pollution—and climate change [27], but also by abandonment in less productive regions [28], especially in densely inhabited lowland areas, such as the Po Plain [29,30]. Among them, there is a relevant role for terricolous lichen diversity in acidic dry grasslands [6,7,10,24,25,26,31], which develop on acidic, mineral, shallow, and oligotrophic soils [32]. In this habitat, terricolous lichen communities are influenced by small-scale disturbance, e.g., by trampling or invasive species, and by climate features [8,10].

This study aims at exploring the relationships between *Cladonia* functional traits and environmental factors that drive community composition in lowland acidic dry grasslands. We hypothesized that (i) the abundance of different functional traits, such as growth forms, reproductive strategies, and secondary metabolites, can be shaped by the main environmental factors, i.e., vegetation dynamics, disturbance, substrate pH, and climatic features (temperature and precipitation); and that (ii) discriminating traits like growth forms and reproduction strategy in detail could give better insights on the responses of these lichens to environmental factors.

## 2. Materials and Methods

### 2.1. Study Area

**Figure 1:**
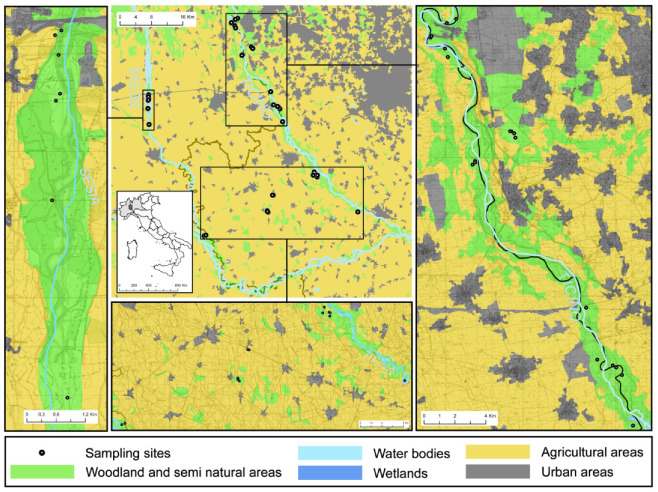
Study area and study sites.

The study was carried out in the central-western Po Plain (northern Italy), in an area located on the two sides of the boundary between the regions of Lombardy and Piedmont (Figure 1). The mean annual temperature ranges between 13.3 and 14.2 °C. Annual rainfall ranges between 788 and 1104 mm. The altitude varies between 61 and 189 m a.s.l.

In this area, 41 sites with lichen-rich acidic dry grasslands were searched for and located (Figure 1). They were clustered along the course of two main rivers, i.e., the Sesia (7 sites) and the Ticino (26 sites), in a stretch of the Po river where the substrate is acidic (3 sites) and in a small area in Lomellina in which residual inland sand dunes still occur (5 sites). These grasslands are attributed to the Natura 2000 Habitat 2330 (“Inland dunes with open *Corynephorus* and *Agrostis* grasslands”) or to a pioneer and acidic facies of Habitat 6210 (“Seminatural dry grasslands and scrubland facies on calcareous substrates”). Acidic dry grasslands are severely threatened in Europe [27,32], and therefore these grasslands have a relevant conservation value. Habitat 2330 has also a biogeographical value in this area since it is at the southernmost edge of its distribution range [30].

In the study area, these grasslands are often fragmented and located in marginal and unproductive areas that are not actively managed and are sometimes degraded due to human disturbance—typically uncontrolled grazing and motorbike riders—with the colonization of invasive species [29,33]. Nevertheless, they frequently host terricolous lichen communities, and though species-poorer than in similar habitats in central Europe, they include some species with a more Mediterranean distribution pattern [10,25,26,34,35].

### 2.2. Sampling

At each site, a linear transect connecting the two furthest vertices of the grassland was laid out. Along each transect, from 1 to 7 circular plots with a 3 m radius were placed at regular intervals proportionally to the site area. This resulted in a total of 122 plots.

Vegetation was recorded in each plot between April and June 2016. We recorded the cover (%) and the mean height (cm) of the five vegetation layers—cryptogamic, herbaceous, lower-shrubby (shrubs up to 1.5 m), higher-shrubby (shrubs between 1.5 and 3 m), arboreal (woody species over 3 m high)—and the cover (%) of each vascular plant, lichen and bryophyte species. Easily recognizable species were identified in the field, whereas difficult specimens were collected and identified in the laboratory. All the specimens are retained in the first author’s personal herbarium.

In each plot, we also recorded the occurrence of disturbance that could impact lichens. Human trampling was estimated according to a categorical scale: 0 (no trampling), 1 (<5 m^2^ showing evidence of trampling on vegetation), 2 (5–10 m^2^), 3 (10–15 m^2^), 4 (>15 m^2^). The impact by lagomorphs was estimated using the abundance of fecal pellets as a proxy [33], according to a categorical scale: 0 (no pellet), 1 (<2 pellet/m^2^), 2 (3–5 pellet/m^2^), 3 (>5 pellet/m^2^). Additionally, soil pH was recorded on the field by means of a portable kit.

### 2.3. The Genus Cladonia

*Cladonia* (Hill.) P. Browne (Cladoniaceae, Lecanorales, Ascomycetes) is a cosmopolitan and megadiverse genus with a wide altitudinal and ecological range, most species being terricolous and ranging from mineral to humus-rich soils [11,12]. *Cladonia* species are characterized by a thallus composed of two parts: a squamulose or crustose primary thallus and a fruticose secondary thallus with a very variable morphology that can include stick-shaped, club-shaped, cup-shaped, sparingly branched or richly branched structures called podetia [11,12,13]. The primary thallus can be ephemeral or persistent, and, in this case, even dominant; on the other hand, the secondary thallus, which is often dominant, in some species can be inconspicuous or even absent [11,12,13].

Reproduction can be sexual or asexual. *Cladonia* has biatorine apothecia which produce simple ascospores; apothecia develop at the tips of capitiform, bacillary and branched podetia, on the edges of scyphipherous podetia or, in few species, directly on primary squamules [11,12,13]. Vegetative propagules can include soredia, schizidia, blastidia, and microsquamules [11,12], but also thallus fragments can act efficiently as propagules [16,36]. Vegetative reproduction involving only the mycobiont is carried out by conidia; conidia are produced in pycnidia, which in *Cladonia* can be located either on the primary squamules or on podetia [11,12,13].

*Cladonia* lichens produce several compounds, chiefly aliphatic acids (e.g., rangiformic and bourgeanic acids), dibenzofurans (e.g., usnic acid), depsides (e.g., homosekikaic and perlatolic acids, atranorin), and depsidones (e.g., fumarprotocetraric, norstictic, and psoromic acids) [37,38,39,40,41,42,43]. Some of them show cytotoxic activity and are much studied for their pharmacological potential [17,18,19], but their ecological roles have been addressed more rarely. More investigated are allelopathic [44,45,46] and anti-herbivorous [47,48,49] effects, rarer are ecological studies that investigated their roles in photoprotection [50,51,52] and in regulating species’ preferences for substrate pH [20,21,22,53].

### 2.4. Functional Traits

Three groups of functional traits were considered: growth form, reproductive strategy, and secondary metabolites.

Previous literature that considered the growth forms of *Cladonia* in the analysis of functional traits used a weak differentiation just between foliose/squamulose (for species without secondary thallus) and fruticose thalli, or between foliose/squamulose, fruticose with simple podetia, and fruticose with branched podetia [6,7,14,15]. The huge diversity occurring within *Cladonia*, not only in morphology but also in size, deserves a sharper and more precise distinction since different shapes and sizes can potentially give different benefits or disadvantages under different environmental conditions, at a microhabitat scale. On the basis of morphological data reported in the main literature sources [11,12,13,54] and many personal observations on the specimens collected for this work, we considered six different growth forms (Table 1).

The reproduction strategy was described by means of the main reproduction type and the position of pycnidia on thallus. The main reproduction type of each species was retrieved from the database ITALIC [55]. Two types were considered: sexual reproduction by ascospores or asexual reproduction by soredia. The position of pycnidia was assessed consulting the main literature sources [11,12,13,54] and also through personal observations on the specimens collected for this work. Two cases were considered: pycnidia on the primary squamules or pycnidia on the podetia. Species with pycnidia on the primary thallus could be expected to have a faster development, being earlier colonizers of pioneer situations, whereas species with pycnidia on podetia could be expected to have slower development and to occur later in the succession since podetia develop after primary thallus.

The occurrence of secondary metabolites was assessed by means of thin-layer chromatography (TLC) performed with the solvents A, B’, and C [56]. The eight most frequent metabolites were atranorin, fumarprotocetraric acid, homosekikaic acid, norstictic acid, perlatolic acid, rangiformic acid, usnic acid, and zeorin. In addition, baeomycesic acid, squamatic acid, and strepsilin occurred only in one rarely recorded species, *Cladonia strepsilis*, and therefore they were not considered in the analysis.

The attribution of the considered functional traits to the 14 *Cladonia* species recorded in the 122 plots is shown in Table 2.

For each trait, the abundance in each plot was calculated as the sum of the abundances of the species with that trait in the plot. The abundances, originally recorded in the field in percent values, were converted in a scale ranging from 1 to 10, as follows: 1–10% = 1; 11–20% = 2; 21–30% = 3; 31–40% = 4; 41–50% = 5; 51–60% = 6; 61–70% = 7; 71–80% = 8; 81–90% = 9; 91–100% = 10.

### 2.5. Environmental Variables

The cover values (%) of the vascular plant biological forms [57] were calculated for each plot based on the floristic composition recorded. Therophytes (annual/biennial herbs), hemicryptophytes, and geophytes (perennial herbs) are part of the herbaceous layer, chamephytes are generally found in the lower-shrubby layer and phanerophytes, typical of higher-shrubby and arboreal layers, can be found in the lower-shrubby layer when young. Biological forms were considered in the analysis as a proxy of vegetation dynamics since it is known that therophytes dominate pioneer stages, also indicating ongoing vegetation dynamics due to disturbance in some cases; hemicryptophytes and geophytes dominate intermediate stages, indicating less active but still ongoing vegetation dynamics; chamephytes and phanerophytes dominate more mature stages, i.e., scrub and forest, indicating the passage from grassland to more developed vegetation types [58].

Climatic variables, i.e., mean annual temperature and annual precipitation (considered as a proxy of humidity), were retrieved for each sampling plot from CHELSA [59].

### 2.6. Data Analysis

To explore the relationships between functional traits and environmental variables, a model-based fourth corner analysis was used. This method is aimed at solving the “fourth corner problem”, by analyzing the relationships between the three matrices (i) species x sites, (ii) species x traits, and (iii) sites x environmental variables, to estimate a matrix with environment-trait associations [60]. In particular, we followed the framework proposed by [61] and implemented it in the R package “mvabund” [62]. This approach proceeds by fitting a GLM with species abundances as a function of species’ traits, environmental variables, and their interactions. The model was fitted using a Poisson distribution with LASSO penalty to enhance prediction accuracy; this latter sets to zero all the coefficient terms that do not explain any variation [61]. In the end, the model was evaluated through diagnostic plots.

## 3. Results

**Figure 2:**
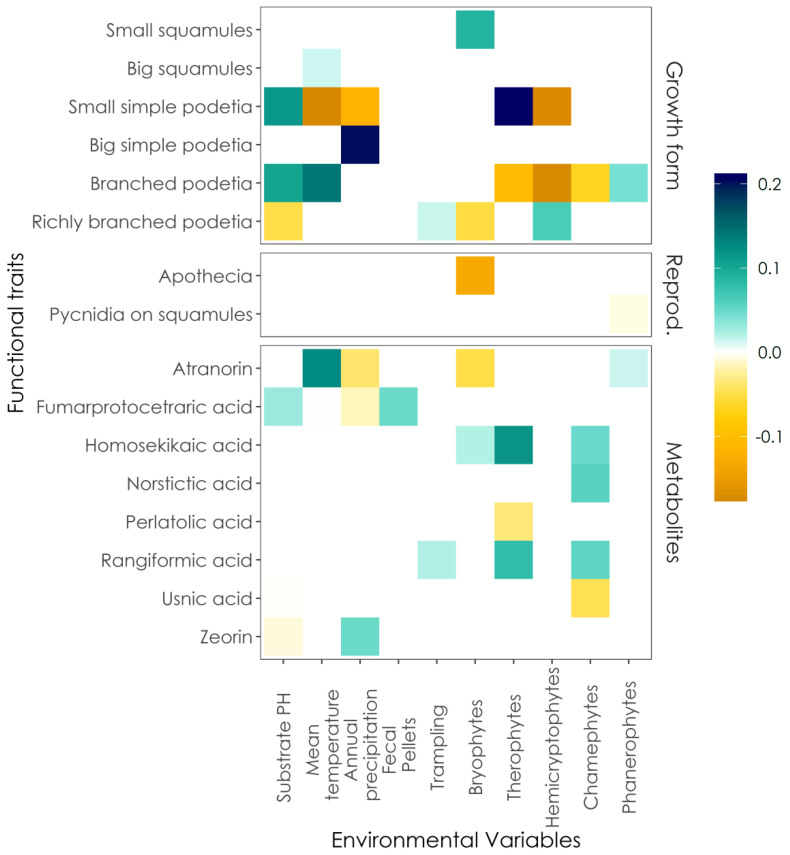
Results of the Fourth Corner Analysis. Statistically significant correlations between traits and environmental variables are represented by colored squares. Darker colors express stronger correlations.

Significant relationships were found between morphological traits and vegetation dynamics, substrate features, disturbance, and climate (Figure 2). Small squamules, small simple podetia, branched, and richly branched podetia correlated with variables describing vegetation dynamics. Big squamules, small and big simple podetia, branched podetia correlated with climatic variables. Small simple podetia, branched and richly branched podetia correlated with substrate pH, and richly branched podetia correlated with trampling.

Reproduction traits were less responsive to environmental factors (Figure 2). Only sexual reproduction and pycnidia located on primary squamules correlated with few variables describing vegetation dynamics.

Significant relationships were found also between secondary metabolites and various predictors associated with vegetation dynamics, substrate, disturbance, and climate (Figure 2). Atranorin, homosekikaic, norstictic, perlatolic, rangiformic, and usnic acids correlated with variables describing vegetation dynamics. Atranorin, fumarprotocetraric acid, and zeorin correlated with climatic variables. Fumarprotocetraric acid and zeorin correlated with substrate pH, while fumarprotocetraric acid correlated with fecal pellets and rangiformic acid with trampling.

## 4. Discussion

In accordance with our hypothesis, morphological, reproduction, and chemical traits of terricolous *Cladonia* species in acid dry grasslands were involved in the responses of these organisms mainly to vegetation dynamics and climate, but also, to a lesser extent, to disturbance and substrate pH.

Some relationships with vegetation dynamics were particularly evident, e.g., species with small simple podetia and species with pycnidia on primary squamules were more frequent in stages dominated by therophytes (pioneer grasslands), decreasing in stages dominated by other biological forms (intermediate-mature grasslands). An opposite pattern was found for species with branched and richly branched podetia and species with perlatolic acid, related to intermediate-mature stages. An allelopathic activity against vascular plants was demonstrated for perlatolic acid [45], but if this was the case, an evident correlation should have been observed for other biological forms considered, not only with therophytes; this negative correlation could better be seen as a link with mature stages of dry grasslands.

Research has suggested photoprotection activity for usnic acid [52] and facilitation in exploiting low light intensities for atranorin [63]. This can explain their correlations with vascular plants which produce a thicker canopy than herbs and forbs, i.e., chamephytes and phanerophytes, which occur in mature stages of dry grasslands. These two metabolites showed opposite patterns in relation to the canopy, i.e., usnic acid correlated negatively and atranorin correlated positively. Considering the 83 *Cladonia* taxa reported in Italy so far [55], none of them contain both these metabolites at the same time [11,12]. This could suggest that the production of one compound instead of another can help these lichens to cope with site-specific light conditions–and, consequently, with a different stage of vegetation succession.

Species with homosekikaic and rangiformic acid correlated positively with the cover of therophytes, and the species which produce at least one of these two metabolites—*C. cariosa*, *C. rangiformis*, *C. rei*, which have different growth forms and reproductive strategies—are widely recognized as early colonizers of prohibitive substrates which often dominate the communities where they develop [13,64,65,66]. However, they also occur, and often dominate in intermediate-mature stages [25,26,64], as backed by the positive correlations with other biological forms of vascular plants. The role of these two compounds in fostering colonization of primitive substrates and long-lasting dominance could be hypothesized.

At a smaller scale, the dynamics of cryptogam communities in dry grasslands are linked also to bryophytes, which are more abundant in intermediate-mature stages [25,26]. However, correlations with bryophyte cover could also suggest facilitation/competition dynamics. The positive relationship with small squamules could be related to water provisioning [67]: for example, inconspicuous *Cladonia* with small squamules could be easier to fit within higher covers of mosses and profit from the water they retain. In contrast, negative correlations with richly branched podetia and reproduction by apothecia suggest competition between mosses and lichens. Richly branched *Cladonia* are typical of intermediate-mature stages of vegetation succession, in which bryophytes also reach high cover values. A high bryophyte cover can make it more difficult for spores to encounter a photobiont for the regeneration of a new lichen thallus.

Competition with bryophytes and vascular plants is not the only stressful factor for lichens in dry grasslands, however, we found only a few correlations between traits and disturbance factors. Trampling is regarded as the main threat to *Cladonia* lichens [68], but the positive correlation with richly branched podetia suggests that a moderate trampling could be a positive factor in open habitats, e.g., as a major driver of dispersal [16,36,69]. Trampling has the positive effect of producing and dispersing thallus fragments and, therefore, it could be particularly beneficial for lichens with large and fragile thalli. Additionally, the abundance of fecal pellets by lagomorphs can have different effects on lichens [33]. In our case, their positive correlation with fumarprotocetraric acid could suggest that this metabolite allows lichens to deal with a nitrified substrate since it is already known that this compound helps in tolerating substrates containing heavy metals [70] and has an antimicrobial activity [71].

Substrate pH is a limiting factor also. Small simple podetia, branched podetial, and fumarprotocetraric acid correlated positively with pH, suggesting that these traits are fostered by subneutral soils; contrariwise, richly branched podetia and zeorin occurred in very acidic substrates. These growth forms include species ranging from acidic to calcareous substrates, e.g., *C. cariosa*, *C. furcata*, *C. rangiformis*, which is the case also for some species with fumarprotocetraric acid; therefore, these correlations could be spurious and due to the incomplete pH range included in our data, limited to acidic substrates. The correlation with zeorin is backed by [21], who suggested that this metabolite could play a role in the interaction with strongly acidic substrates.

Our results indicate that climatic factors may also contribute to the selection of species traits in local communities, determining species dynamics and community composition in a climate change scenario [8,72,73]. An outstanding example is that of species with atranorin, which may be fostered by increasing temperatures and decreasing precipitation. The size of simple podetia increased with increasing precipitation, with small simple podetia correlating negatively and large simple podetia correlating positively, which could be related to a better water exploitation capacity by larger podetia [74]; however, small simple podetia can also be expected to decrease at increasing temperatures. Partial responses were found also for species with big squamules and fumarprotocetraric acid, expected to increase respectively at increasing temperatures and decreasing precipitations, and by species with big simple podetia and zeorin, expected to decrease with decreasing precipitation.

## 5. Conclusions

*Cladonia* shows a wide variety of thallus growth forms, reproductive strategies, and chemotypes [11,12], being, therefore, a suitable model genus to assess the relationships between environmental factors and species traits of terricolous communities. Therefore, our results can have broader applicability for a better understanding of these communities from a functional standpoint, also considering that *Cladonia*-dominated communities often have a similar composition across different biogeographical contexts (cf. [11,12,55]).

The relationships between species traits and vegetation dynamics can help elucidate their colonization patterns in open dry habitats, also reflecting the effect of biotic interactions between lichens and bryophytes or vascular plants. Furthermore, the correlations of some metabolites with certain environmental factors could help in addressing future research aimed at understanding their ecological roles, which are still largely unexplored.

From a conservation standpoint, the contrasting relationships between some traits and vegetation dynamics support the view that management of acidic dry grasslands should aim at maintaining patches at different dynamics stages (pioneer, intermediate, mature) to maximize taxonomic and functional diversity of lichen communities [10].

## Figures and Tables

**Table 1:** Growth forms of genus *Cladonia* considered in this study.

Abbreviation	Description
Small squamules	Squamulose thallus with small squamules: species usually without podetia and with squamules usually shorter than 5 mm; e.g., *Cladonia strepsilis*.
Big squamules	Squamulose thallus with big squamules: species usually without podetia and with squamules usually longer than 5 mm; e.g., *Cladonia foliacea*.
Small simple podetia	Thallus with small simple podetia: species with persistent primary thallus and usually with bacillar or capitiform podetia shorter than 10 mm; e.g., *Cladonia peziziformis*.
Big simple podetia	Thallus with big simple podetia: species with persistent or ephemeral primary thallus and usually with bacillar of scyphipherous podetia taller than 10 mm; e.g., *Cladonia pyxidata*, *Cladonia rei*.
Branched podetia	Thallus with branched podetia: species with ephemeral primary thallus and with sparingly branched podetia; e.g., *Cladonia furcata*.
Richly branched podetia	Thallus with richly branched podetia: species with ephemeral primary thallus and with richly branched, bush-shaped podetia; e.g., *Cladonia rangiformis* and species belonging to subgenus *Cladina*.

**Table 2:** Functional traits of the 14 *Cladonia* species recorded in the 122 plots. Nomenclature follows Nimis and Martellos (2020).

Species	Growth Form	Position of Pycnidia	Reproduction	Metabolites
*Cladonia cariosa* (Ach.) Spreng.	Small simple podetia	Squamules	Spores	Atranorin, rangiformic acid
*Cladonia chlorophaea* (Sommerf.) Spreng.	Big simple podetia	Podetia	Soredia	Fumarprotocetraric acid
*Cladonia coccifera* (L.) Willd.	Big simple podetia	Podetia	Spores	Usnic acid, zeorin
*Cladonia fimbriata* (L.) Fr.	Big simple podetia	Podetia	Soredia	Fumarprotocetraric acid
*Cladonia foliacea* (Huds.) Willd.	Big squamules	Squamules	Spores	Fumarprotocetraric acid, usnic acid
*Cladonia furcata* (Huds.) Schrad.	Branched podetia	Podetia	Spores	Atranorin, fumarprotocetraric acid
*Cladonia peziziformis* (With.) J.R.Laundon	Small simple podetia	Squamules	Spores	Fumarprotocetraric acid
*Cladonia polycarpoides* Nyl.	Big squamules	Squamules	Spores	Norstictic acid
*Cladonia portentosa* (Dufour) Coem.	Richly branched podetia	Podetia	Spores	Perlatolic acid, usnic acid
*Cladonia pyxidata* (L.) Hoffm.	Big simple podetia	Podetia	Spores	Fumarprotocetraric acid
*Cladonia rangiformis* Hoffm.	Richly branched podetia	Podetia	Spores	Atranorin, rangiformic acid
*Cladonia rei* Schaer.	Big simple podetia	Podetia	Soredia	Fumarprotocetraric acid, homosekikaic acid
*Cladonia strepsilis* (Ach.) Grognot	Small squamules	Squamules	Spores	Baeomycesic acid, squamatic acid, strepsilin
*Cladonia uncialis* (L.) F.H.Wigg.	Branched podetia	Podetia	Spores	Usnic acid

## Data Availability

The data presented in this study are available on request from the corresponding author.
